# Retroperitoneal, Liver, and Lymph Node Metastasis from Proximal Tibial Osteosarcoma: A Case Report

**DOI:** 10.1055/s-0041-1731360

**Published:** 2021-10-13

**Authors:** Daniela Gutierrez, Carlos A. Sánchez, Francisco B. Linares, Oscar Messa

**Affiliations:** 1 Departamento de Ortopedia e Traumatologia, Pontificia Universidad Javeriana, Bogotá, Colômbia; 2 Departamento de Ortopedia e Traumatologia, Pontificia Universidad Javeriana, Hospital Universitario San Ignacio, Bogotá, Colômbia; 3 Departamento de Oncologia Ortopédica, Pontificia Universidad Javeriana, Hospital Universitario San Ignacio, Bogotá, Colômbia; 4 Departamento de Patologia, Pontificia Universidad Javeriana, Hospital Universitario San Ignacio, Bogotá, Colômbia

**Keywords:** lymphatic metastasis, metástasis, osteosarcoma, tibia

## Abstract

The present study describes the case of a male adult with an osteosarcoma in the proximal tibia, treated with limb salvage with endoprosthesis and chemotherapy. The patient developed an unusual metastatic pattern compromising the liver, bone, and inguinal lymph nodes, without local recurrence in the tibia or pulmonary metastases. Osteosarcoma (OS) is the second most frequent primary bone tumor after multiple myeloma in adults. Frequent sites of metastases in case of disease progression are the lungs and bone. Extrapulmonary metastases are rare. The development of new schemes of chemotherapy have improved life expectancy in osteosarcoma patients but have also altered the usual patterns of metastases, resulting in unusual metastatic locations.

## Introduction

Advances in the development of new chemotherapy and radiotherapy schemes, as well as in orthopedic oncologic surgery and prognosis of patients with osteosarcoma (OS) have improved survival from 15 to more than 60%. 1 2 However, approximately 40% of patients relapse and present disease progression with frequent metastases to the lungs and bone. 2 Other sites of secondary compromise, such as abdominal organs, lymph nodes, or retroperitoneum, are rare. Despite the advances in treatment options, the prognosis of patients with unusual metastases remains grim, with high mortality rates. The use of new chemotherapy regimens may be associated with the induction of atypical patterns of spread and disease progression, possibly by favoring the selection of different phenotypes in tumor cells. 2 We present the case of an adult male with unusual metastatic involvement of osteosarcoma, caused by a large retroperitoneal mass, an inguinal lymph node conglomerate, with liver and bone metastases without local or pulmonary recurrence.

## Case Report

**Fig. 1:**
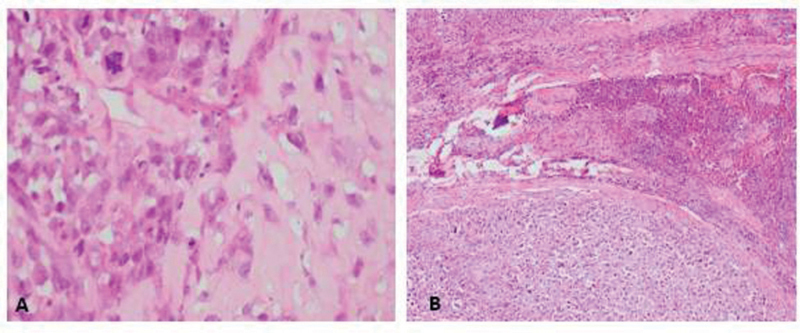
( **A** ) H/E 40x. Malignant tumor with osteoid production corresponding to an osteosarcoma. ( **B** ) H/E 40x. Lymph node metastases of osteosarcoma. Sheets of pleomorphic cells with extensive necrosis, abundant mitoses and osteoid.

A 46-year-old male presented with pain in the left knee radiating distally to the leg. X-ray and magnetic resonance studies exhibited a lytic lesion of the proximal tibia that required an open biopsy, which confirmed osteosarcoma ( Fig. 1A ). Computed tomography scans of the thorax, abdomen and pelvis were negative for metastatic lesions. Neoadjuvant treatment began with 3 cycles of doxorubicin and cisplatin, and, 6 months later, limb salvage surgery was held with complete resection of the tumor and reconstruction with a proximal tibial endoprosthesis. Histopathology confirmed a proximal tibial OS, with tumor-free margins, and a 30% viable tumor, indicating a grade II A response to neoadjuvant management.

Subsequently, the patient completed three cycles of adjuvant chemotherapy with the previous scheme, with adequate tolerance to treatment and without toxicity. One year after the surgical procedure, he presented episodes of limb lymphedema and pain. Doppler ultrasound ruled out deep vein thrombosis, and an abdominal CAT scan disclosed inguinal and iliac lymphadenopathies. Chest CAT scan revealed a non-specific pulmonary nodule smaller than 1 cm. At the time, thoracic surgery service did not consider it as a metastatic lesion. Later, an inguinal node biopsy was performed, and the histopathological analysis confirmed a malignant tumor and OS metastases ( Fig. 1B ).

Considering that the patient presented with a relapse in the lymph nodes and inguinal soft tissues, without local recurrence at the initial tumor site, resection of the metastasis was decided upon. During the intervention, a solid mass of approximately 10 cm in diameter was evidenced in the left inguinal region adhered to the lateral aspect of the bladder, the external iliac vessels, the spermatic chord, and the inguinal ligament. It presented with an extensive involvement of the inguinal nodes, so a left inguinoiliac lymph node dissection was performed, achieving an R2 resection of the mass (given the compromise of the vascular structures and extensive soft-tissue fibrosis). The histopathological study identified multiple conglomerate nodal structures of 5 to 6 cm in diameter, considered to be metastases with massive angioinvasion and extension to soft tissues. After this intervention, palliative chemotherapy was held using gemcitabine and docetaxel, of which the patient received 1 cycle of treatment.

**Fig. 2:**
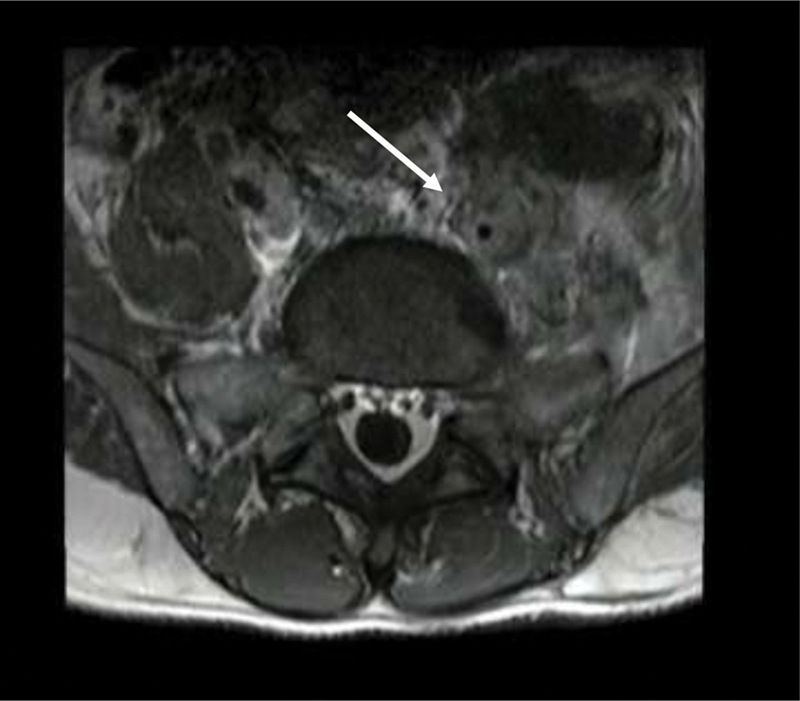
Magnetic resonance of the lumbosacral spine showing a mass in the paravertebral soft tissues, infiltrating the body of the L5 vertebra (arrow).

One month later, the patient presented with severe pain in the lower left limb as well as monoplegia, acute renal failure, and hematuria. A magnetic resonance imaging (MRI) of the lumbosacral spine revealed an irregular left anterolateral paravertebral mass in the lower lumbar segment that infiltrated the psoas muscle and the anterior cortex of the L5 vertebral body ( Fig. 2 ).

**Fig. 3:**
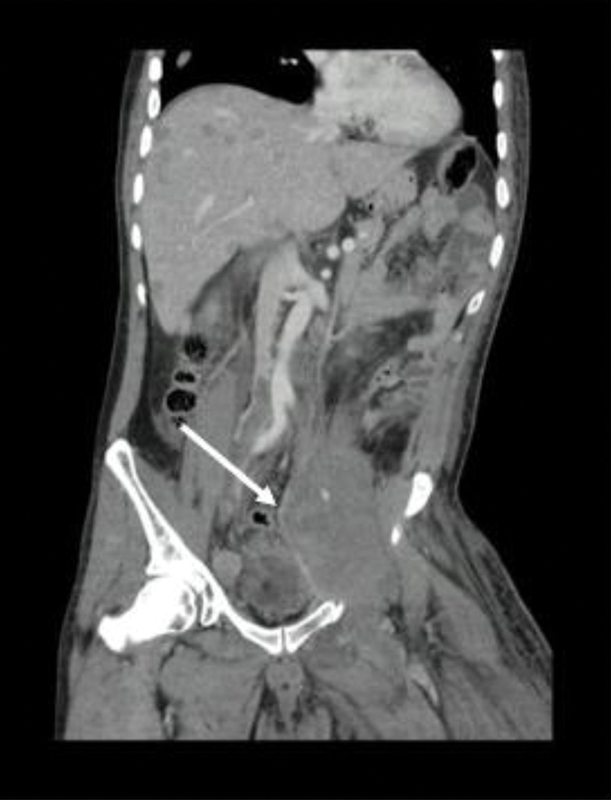
Thoracoabdominal CT in a coronal view, with a soft tissue mass infiltrating the left psoas muscle extending to the inguinal region (arrow).

**Fig. 4:**
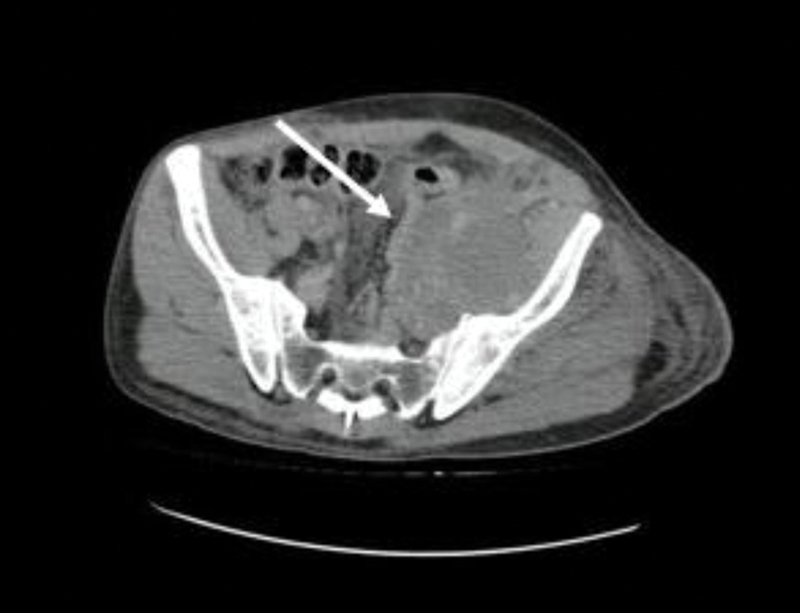
Axial view of an abdominal CT, with a soft tissue mass infiltrating the left psoas muscle (arrow).

In addition, the contrasted thoracoabdominal CAT scan identified multiple hypodense lesions compatible with metastases in the liver parenchyma, pelvic bones, and a mass of approximately 140 × 78 mm on the left iliac psoas muscle that extended from L3 to the inguinal region and surrounded the internal and external iliac vessels. Additionally, para-aortic, inter aorto-cava, and right external iliac lymph nodes were found ( Figs. 3 and 4 ).

**Fig. 5:**
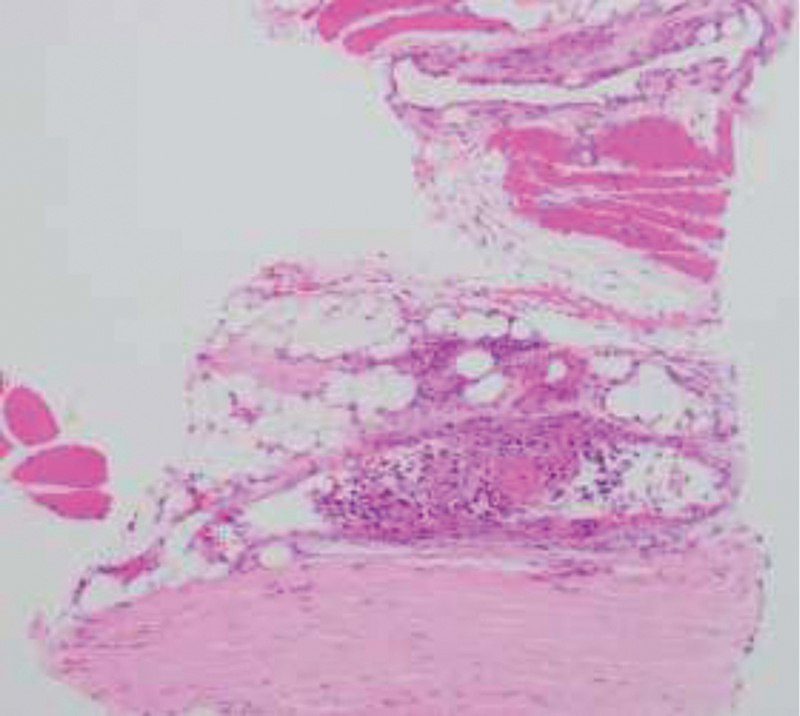
H8/e 10x. Neoplasic thrombi with osteoid matrix amongst malignant cells compatible with metastatic osteosarcoma.

A CAT-guided biopsy of this mass was performed, showing metastatic involvement due to a high-grade osteosarcoma ( Fig. 5 ).

The patient persisted with clinical deterioration, developed deep vein thrombosis in the lower left limb and acute pulmonary thromboembolism, persistent hematuria, and dehydration, after which he died, 2 years after the initial diagnosis of OS.

## Discussion

The occurrence of metastasis is part of the natural course of osteosarcoma (OS), with pulmonary metastasis being the most frequent. 1 3 4 5 6 7 Using chemotherapy as part of the integral management, a change in the usual patterns of metastasis has been described, with respect to what was reported in 1975 by Jeffree et al. 8 Although this may be the result of better identification and reporting of new cases of OS, the improvement in survival of patients with OS has revealed new patterns of extra-pulmonary metastasis, particularly in the peritoneum and lymph nodes. 1 2 3 6 7 9 The literature is not clear regarding the epidemiology of lymphatic metastases in OS, and most of the available information comes from case reports and case series, with an incidence between 4 and 11%. 1 6 7 9 10 The presence of micrometastases that can achieve a dissemination of OS has also been described, which could impact the survival rates. 4

One of the most important aspects that impact survival in patients with OS is the development of metastases. 1 8 9 10 It seems that the presence of certain metastases could be associated with more aggressive OS variants. 1 6 7 8 9 10 It has been hypothesized that this change in the usual pattern of extrapulmonary metastasis is the result of the use of more efficient chemotherapies that target certain tumor cells, leaving room for more aggressive variants with greater metastatic potential. 2 3 4 5 9 10 This could explain the outcome of lymph node and abdominal metastases (especially retroperitoneal) that are associated with difficult-to-manage relapse and high mortality at the time of diagnosis. 2 3 9 10

The present case describes an aggressive and difficult-to-treat type of bone tumor, with a poor response to chemotherapy, with distant recurrence, and, eventually, a fatal outcome. Although the presence of lymph node metastases is exotic, the occurrence of extrapulmonary metastases is increasing, and, therefore, it is essential to understand the scope of a tumor that science may not have fully understood yet.
