# Supplementary material for: Retroperitoneal, Liver, and Lymph Node Metastasis from Proximal Tibial Osteosarcoma: A Case Report
Source: Rev Bras Ortop (Sao Paulo). 2021 Oct 13;59(4):e637–40. doi: 10.1055/s-0041-1731360 (PMC11374403; doi:10.1055/s-0041-1731360)
Supplement: Related texts

# Research article: Metástase retroperitoneal, hepática e de linfonodo de osteossarcoma tibial proximal: Relato de caso

**Keywords:** metástase, metástase linfática, osteossarcoma, tíbia

## Abstract

Homem adulto com osteossarcoma na tíbia proximal, tratado com cirurgia de salvamento de membro com endoprótese e quimioterapia. Ele desenvolveu um padrão metastático incomum, comprometendo fígado, ossos e linfonodos inguinais, sem recorrência local na tíbia nem metástases pulmonares. O osteossarcoma (OS) é o segundo tumor ósseo primário mais frequente, depois do mieloma múltiplo em adultos. Os locais frequentes das metástases, em caso de progressão da doença são os pulmões e os ossos. As metástases extrapulmonares são raras. O desenvolvimento de novos esquemas de quimioterapia melhorou a expectativa de vida dos pacientes com osteossarcoma, porém, alterou também os padrões usuais de metástases, resultando em localizações metastáticas incomuns.

## Introdução

Os avanços no desenvolvimento de novos esquemas de quimioterapia e radioterapia, assim como os avanços nas cirurgias ortopédicas oncológicas, melhorou o prognóstico dos pacientes com osteossarcoma (OS), aumentando a sobrevida de 15% para mais de 60%. 1 2 No entanto, aproximadamente 40% dos pacientes apresentam recidiva e progressão da doença com metástases frequentes para os pulmões e ossos. 2 Outros locais de comprometimento secundário, como os órgãos abdominais, linfonodos ou retroperitônio são raros. Apesar dos avanços nas opções de tratamento, o prognóstico dos pacientes com metástases incomuns permanece sombrio, com altas taxas de mortalidade. O uso de novos esquemas de quimioterapia pode estar associado à indução de padrões atípicos de disseminação e progressão da doença, possivelmente por favorecer a seleção de diferentes fenótipos das células tumorais. 2 Apresentamos o caso de um homem adulto com envolvimento metastático incomum de osteossarcoma, causado por uma grande massa retroperitoneal, um conglomerado de linfonodos inguinais, com metástases hepáticas e ósseas sem recorrência local ou pulmonar.

## Relato de Caso

Um homem de 46 anos de idade consultou-se por causa de dor no joelho esquerdo, com irradiação distal para a perna. Os estudos de raios-X e ressonância magnética exibiram uma lesão lítica da tíbia proximal, exigindo uma biópsia aberta confirmando o osteossarcoma ( Fig. 1A ). As tomografias do tórax, abdômen e pelve foram negativas para lesões metastáticas. O tratamento neoadjuvante foi iniciado com 3 ciclos de doxorrubicina e cisplatina, 6 meses após a cirurgia de salvamento do membro com ressecção completa do tumor e reconstrução com endoprótese tibial proximal. A histopatologia confirmou um OS tibial proximal, com margens livres de tumor, e 30% de tumor viável, indicando uma resposta de grau II A ao tratamento neoadjuvante.

**Fig. 1:** ( **A** ) H/E 40x. Tumor maligno com produção de osteoide correspondendo a um osteossarcoma. ( **B** ) H/E 40x. Metástases de linfonodo de OS. Lâminas de células pleomórficas com necrose extensa, mitose e osteoide abundante.

Posteriormente, o paciente completou 3 ciclos de quimioterapia adjuvante com o esquema anterior, apresentando adequada tolerância ao tratamento e sem toxicidade. Após 1 ano do procedimento cirúrgico, apresentou episódios de linfedema do membro e dor. A ultrassonografia Doppler descartou trombose venosa profunda e a TC abdominal revelou linfadenopatias inguinais e ilíacas. A TC do tórax revelou um nódulo pulmonar inespecífico menor que 1 cm. Na época, o serviço de cirurgia torácica não considerava como uma lesão metastática. Posteriormente, foi realizada biópsia do linfonodo inguinal, a análise histopatológica confirmou tumor maligno e metástases de OS ( Fig. 1B ).

Considerando que o paciente apresentava recidiva dos linfonodos e das partes moles inguinais, sem recidiva local no sítio inicial do tumor, optou-se pela ressecção da metástase. Durante a intervenção, foi evidenciada uma massa sólida de aproximadamente 10 cm de diâmetro na região inguinal esquerda, aderida à face lateral da bexiga, aos vasos ilíacos externos, ao cordão espermático e ao ligamento inguinal. Apresentava extenso comprometimento dos linfonodos inguinais, sendo realizada a dissecção linfonodo inguinoilíaca esquerda, obtendo-se uma ressecção R2 da massa (devido ao comprometimento das estruturas vasculares e da extensa fibrose das partes moles). O estudo histopatológico identificou múltiplas estruturas nodais conglomeradas de 5 a 6 cm de diâmetro, consideradas metástases com grande angioinvasão e extensão para os tecidos moles. Após essa intervenção, foi realizada uma quimioterapia paliativa com a administração dos fármacos gencitabina e docetaxel com 1 ciclo de tratamento.

Um mês depois, o paciente apresentou dor intensa no membro inferior esquerdo, além de monoplegia, insuficiência renal aguda e hematúria. Uma ressonância magnética da coluna lombossacra, revelou uma massa paravertebral anterolateral esquerda irregular, no segmento lombar inferior que infiltrava o músculo psoas e o córtex anterior do corpo vertebral L5 ( Fig. 2 ).

**Fig. 2:** Ressonância magnética da coluna lombossacra, evidenciando uma massa nos tecidos moles paravertebrais, infiltrando o corpo da vértebra L5 (seta).

Além disso, a TC tóraco-abdominal contrastada identificou múltiplas lesões hipodensas compatíveis com metástases no parênquima hepático, ossos pélvicos e uma massa de aproximadamente 140 × 78 mm no músculo psoas ilíaco esquerdo, se estendendo da L3 à região inguinal, circundando os vasos ilíacos interno e externo. Além disso, foram encontrados linfonodos para-aórticos, inter-aorto-cava e ilíaco externo direito ( Figs. 3 e 4 ).

**Fig. 3:** TC tóraco-abdominal em corte coronal, com uma massa de tecido mole infiltrando o músculo psoas esquerdo com extensão para região inguinal (seta).

**Fig. 4:** TC abdominal em corte axial, com uma massa de tecido mole infiltrando o músculo psoas esquerdo (seta).

Foi realizada biópsia dessa massa guiada por TAC, evidenciando envolvimento metastático devido a um osteossarcoma de alto grau ( Fig. 5 ).

**Fig. 5:** H8/e 10x. Trombos neoplásicos com matriz osteoide entre células malignas compatíveis com OS metastático.

O paciente persistiu com agravamento clínico, evoluiu com trombose venosa profunda no membro inferior esquerdo e tromboembolismo pulmonar agudo, hematúria persistente e desidratação, falecendo dois anos depois do diagnóstico inicial de OS.

## Discussão

A ocorrência de metástases faz parte do curso natural do osteossarcoma (OS), sendo a metástase pulmonar a mais frequente. 1 3 4 5 6 7 Usando a quimioterapia como parte do tratamento integral, em relação ao que foi informado em 1975 por Jeffree et al, 8 houve uma mudança nos padrões usuais da metástase. Embora isso possa ser o resultado de uma melhor identificação e também do relato de novos casos de OS, a melhora na sobrevida dos pacientes com OS revelou novos padrões de metástases extrapulmonares, particularmente no peritônio e linfonodos. 1 2 3 6 7 9 A literatura não é clara a respeito da epidemiologia das metástases linfáticas em OS, a maioria das informações disponíveis vem de relatos de casos e séries de casos, com uma incidência entre 4% e 11%. 1 6 7 9 10 A presença de micrometástases que podem levar à disseminação de OS também foi descrita, o que poderia impactar nas taxas de sobrevida. 4

Um dos aspectos mais importantes que afetam a sobrevida dos pacientes com OS, é o desenvolvimento de metástases. 1 8 9 10 Parece que a presença de certas metástases poderia estar associada a variantes mais agressivas do OS. 1 6 7 8 9 10 Presume-se que essa mudança no padrão usual da metástase extrapulmonar, seja o resultado do uso de quimioterapias mais eficientes que visam certas células tumorais, deixando espaço para variantes mais agressivas com maior potencial metastático. 2 3 4 5 9 10 Isso poderia explicar o resultado de linfonodo e metástases abdominais (especialmente as retroperitoneais) que estão associadas à recidivas de difícil controle e alta mortalidade no momento do diagnóstico. 2 3 9 10

Este caso descreve um tipo de tumor ósseo agressivo e difícil de tratar, apresentando uma baixa taxa de resposta à quimioterapia, com recorrência distante, eventualmente, com desfecho fatal. Embora a presença de metástases em linfonodos seja incomum, a ocorrência de metástases extrapulmonares está aumentando, portanto, é essencial entender o escopo de um tumor que a ciência pode não ter totalmente compreendido.
